# Early Blood Clot Detection Using Forward Scattering Light Measurements Is Not Superior to Delta Pressure Measurements

**DOI:** 10.3390/bios13121012

**Published:** 2023-12-04

**Authors:** Anna Fischbach, Michael Lamberti, Julia Alexandra Simons, Erik Wrede, Alexander Theißen, Patrick Winnersbach, Rolf Rossaint, André Stollenwerk, Christian Bleilevens

**Affiliations:** 1 Department of Anesthesiology, University Hospital RWTH Aachen, Pauwelsstraße 30, 52074 Aachen, Germany; alexandra.simons@rwth-aachen.de (J.A.S.); atheissen@ukaachen.de (A.T.); pwinnersbach@ukaachen.de (P.W.); rrossaint@ukaachen.de (R.R.); 2 Informatics 11—Embedded Software, RWTH Aachen University, 52074 Aachen, Germany; lamberti@embedded.rwth-aachen.de (M.L.); erik.wrede@rwth-aachen.de (E.W.); stollenwerk@embedded.rwth-aachen.de (A.S.)

**Keywords:** forward scattered light measurement, sensor, membrane oxygenator, blood clot detection, in vitro test

## Abstract

The occurrence of thrombus formation within an extracorporeal membrane oxygenator is a common complication during extracorporeal membrane oxygenation therapy and can rapidly result in a life-threatening situation due to arterial thromboembolism, causing stroke, pulmonary embolism, and limb ischemia in the patient. The standard clinical practice is to monitor the pressure at the inlet and outlet of oxygenators, indicating fulminant, obstructive clot formation indicated by an increasing pressure difference (ΔP). However, smaller blood clots at early stages are not detectable. Therefore, there is an unmet need for sensors that can detect blood clots at an early stage to minimize the associated thromboembolic risks for patients. This study aimed to evaluate if forward scattered light (FSL) measurements can be used for early blood clot detection and if it is superior to the current clinical gold standard (pressure measurements). A miniaturized in vitro test circuit, including a custom-made test chamber, was used. Heparinized human whole blood was circulated through the test circuit until clot formation occurred. Four LEDs and four photodiodes were placed along the sidewall of the test chamber in different positions for FSL measurements. The pressure monitor was connected to the inlet and the outlet to detect changes in ΔP across the test chamber. Despite several modifications in the LED positions on the test chamber, the FSL measurements could not reliably detect a blood clot within the in vitro test circuit, although the pressure measurements used as the current clinical gold standard detected fulminant clot formation in 11 independent experiments.

## 1. Introduction

Extracorporeal membrane oxygenation (ECMO) temporarily supports patients with respiratory or cardiac failure [1]. It provides oxygenation, carbon dioxide removal, and circulatory support [2]. Over the last decade, the use of extracorporeal life support for adults with acute respiratory failure has significantly increased, especially during the influenza (H1N1) pandemic in 2009 [3] and the COVID-19 pandemic [4].

Despite its increasing use, ECMO therapy is associated with several complications [5]: bleeding [6,7], hemolysis [8,9], thrombocytopenia [10], infections, thrombosis, and thromboembolisms [11,12]. The incidence of thrombus formation with or without embolization was found to be as high as 53% for veno-venous ECMO therapy [13].

Thrombus formation within a membrane oxygenator can results in a life-threatening situation due to arterial thromboembolism, causing stroke, pulmonary embolism, and limb ischemia in the patient. Hence, a prompt replacement of the ECMO circuit is necessary, requiring a highly qualified clinical team.

As the standard monitoring parameter, the pressure gradient across the membrane oxygenator is continuously measured (ΔP). A rise in the pressure gradient indicates a possible occurrence of a blood clot within the membrane oxygenator.

However, there is no linear correlation between the size of a blood clot and the pressure difference [14]. Consequently, the blood clot might be detected too late and might have already caused a life-threatening situation for the patient [15,16].

Due to these reasons, a more sensitive method other than the measurement of the pressure gradient is necessary to detect a blood clot at an early stage.

The hemoglobin (Hb) of erythrocytes absorbs and reflects light [14]. During the formation of a blood clot, the erythrocytes are trapped within a fibrin net. As a result, the hematocrit decreases in the area surrounding the clot, while it increases within the thrombus [17,18]. This change in hematocrit leads to a change in light absorption and scattering. Morita et al. [18] used this observation to detect a blood clot in a blood pump within an in vitro ECMO circuit. They employed light-emitting diodes (LEDs) positioned on one side of the blood pump and photodiodes on the same side to measure changes in the light intensity of backward scattered light (BSL). Their study evaluated the feasibility of using forward scattered light (FSL) measurements rather than BSL measurements for early blood clot detection.

## 2. Materials and Methods

**Figure 1:**
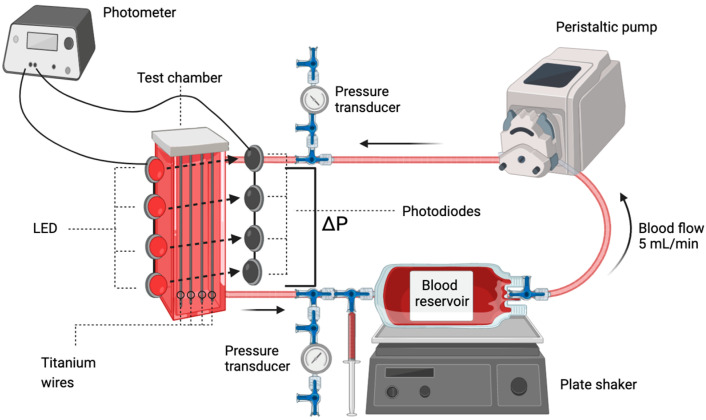
Illustration of the in vitro test circuit. ΔP: pressure difference across the test chamber. Blood samples were withdrawn with a syringe before the blood entered the blood reservoir. This test circuit was adapted from a previous study [19], and slight adjustments were made.

**Figure 2:**
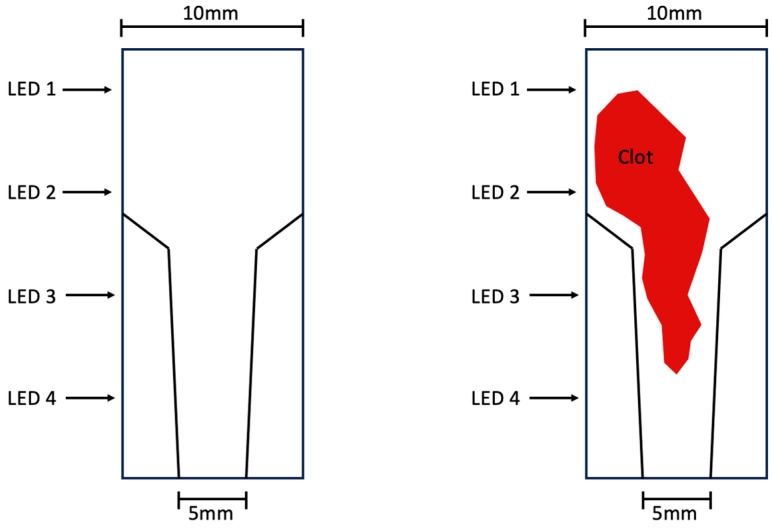
Position of LEDs 1–4 on the test chamber.

The in vitro circuit was adapted from a previous study [19] and slight adjustments were made. Heparinized human whole blood was circulated at a blood flow rate of 5 mL/min from the blood reservoir through a roller pump in an enclosed cuvette and back into the blood reservoir (Figure 1). Four LEDs were positioned on one side of the cuvette, while four photodiodes were placed on the opposite side of the cuvette for light detection (Figure 2). Low heparinized human whole blood (0.6 IU heparin/mL blood) from healthy volunteers was used (approval EK22-355 from the Ethics Committee of the Medical Faculty of RWTH Aachen University). The pressure at the inlet and outlet of the cuvette (ΔP) and the light intensity of the FSL through the cuvette were continuously measured during blood circulation. To provide supplementary coagulation monitoring, both the platelet count (PLT) and the β-thromboglobulin (β-TG) concentration were analyzed at 10 min intervals. After the pressure difference significantly increased (ΔP > 100 mmHg), the measurement was stopped. The blood was carefully flushed out from the system using 50 mL saline to visualize the blood clot within the test system.

A standard transfusion set reservoir (T3986, Fresenius Kabi AG, Bad Homburg, Germany) and a custom-made test chamber were used in this setup. Every test chamber was manufactured by hand based on a semi-micro polystyrene cuvette (67.742, Sarstedt AG, Nümbrecht, Germany). Boreholes were drilled at the base and on the lid of the cuvette to place Titanium wires (ASTM F-67 graded) in the center of the test chamber as a foreign surface in the blood flow path to trigger blood clot formation according to our previous study [19].

Polyethylene infusion lines (1.0 × 2.0 mm, Original Perfusor^®^, 8255059, B. Braun AG, Melsungen, Germany) were inserted into the bottom (blood inlet) and top (blood outlet) of the cuvette.

The connection between the reservoir and test chamber was established using eight 3-way stopcocks (16494C, B. Braun AG, Melsungen, Germany) and PVC tubing (Tygon^®^ ST R-3607, Compagnie de Saint-Gobain S.A., Courbevoie, France) connected to a peristaltic pump (Ismatec 4408 Reglo ICC, Cole-Parmer GmbH, Wertheim, Germany).

Pressure transducers (SP844, Memscap, Skoppum, Norway) were positioned at the inlet and outlet of the test chamber for continuous pressure measurement. The reservoir bag was placed on a plate shaker (MS3B, IKA-Werke GmbH, Staufen, Germany) to minimize the risk of blood clot formation within the bag. The shaker was operated in the continuous shaking mode at 500 rpm. The entire test circuit was placed inside an incubation hood (8863202, Sartorius Lab Instruments GmbH, Göttingen, Germany) to ensure a constant temperature of 37 °C. Heparinized human whole blood circulated through the system during each experiment while the light intensity of the FSL and pressure difference were continuously monitored.

### 2.1. Blood Collection from Human Donors and Filling the Circuit

For each experiment, 75 mL of human whole blood was withdrawn via venipuncture into two 50 mL syringes (8728810F, B. Braun, Melsungen, Germany), pre-primed with heparin sodium (12626723/0319, B. Braun, Melsungen, Germany) at concentrations of 0.6 IE heparin/mL blood (clotting group) and 1.5 IE heparin/mL blood (control group), respectively. The Ethics Committee of the Medical Faculty of RWTH Aachen University approved the blood collection (EK22-355), and informed consent was obtained from the volunteers.

Baseline measurements (BL) were taken by directly drawing blood into a 3 mL citrated sample tube (05.1165, Sarstedt AG, Nümbrecht, Germany) and a 3 mL syringe (4606027V, B. Braun, Melsungen, Germany) for cell count measurements (MEK-6500K, Nihon Kohden, Tokyo, Japan) as well as enzyme-linked immunosorbent assays (ELISAs) for β-TG measurements. In the blood clot formation experiments (clotting group), 0.6 IE heparin/mL blood was used. In experiments without blood clot formation (control group), 1.5 IE heparin/mL blood was used.

Immediately after the blood was withdrawn from the blood donor, the reservoir was filled with 75 mL of heparinized blood while air was carefully removed from the circuit. Once the peristaltic pump was started at a 5 mL/min blood flow rate, the in vitro circuit was filled with blood from the reservoir. After the entire in vitro circuit was primed, the FSL intensity and pressure difference were continuously recorded.

### 2.2. Pressure Measurement

Pressure transducers (SP844, MEMSCAP S.A., Crolles, France) were placed on a transducer pressure dome (840-22, HJK Sensoren + Systeme GmbH & Co. KG, Merching Germany) at the inlet and outlet of the test chamber for continuous pressure measurement. The pressure difference across the test chamber was calculated (ΔP). The pressure was continuously recorded using an amplifying bridge (ML224 Quad Bridge Amplifier, ADInstruments, Dunedin, New Zealand) and a PowerLab data acquisition device (ADInstruments, Dundin, New Zealand). LabChart Pro (Version 8.1.13 2018, ADInstrments, Dunedin, New Zealand) was used for data recording.

### 2.3. Measurement of FSL

Four LEDs (TSHG6200, Vishay Intertechnology, Malvern, PA, USA) with a peak wavelength of 850 nm projected light onto four photodiodes (BPW-34, Vishay Intertechnology, Malvern, PA, USA) with a peak sensitivity of 850 nm. The LEDs and photodiodes were placed in a 3D-printed mount (Prusament PLA—Galaxy Black, Prusa Research, Prague, Czech Republic) that shielded the cuvette from outside light and ensured that the emitted light had to travel through the blood to reach the photodiodes.

The wavelength was chosen due to its near alignment with the isosbestic point of oxyhemoglobin (HbO_2_) and deoxyhemoglobin (Hb) at 810 nm. The light absorption characteristics of Hb and HbO_2_ are equivalent at the wavelength of 810 nm, ensuring minimal influence of blood oxygen saturation changes on measurements [20].

The light intensity measurements were conducted using a custom-built, high-resolution lock-in photometer [21]. The photometer features two input channels for photodiodes and uses the lock-in principle to take precise measurements of small photocurrents. During lock-in measurements, the LEDs are pulsed at a high frequency. For each pulse (t_ON_) and following period where the LED is off (t_OFF_), photocurrents from the photodiodes are integrated to a voltage and then converted into a digital signal (*S*_ON_, *S*_OFF_) using the internal DDC112 ADC (Texas Instruments, Dallas, TX, USA). The measurement result is calculated from the difference between these signals: *S*_result_ = *S*_ON_ − *S*_OFF_. Since *S*_OFF_ includes the photodiode dark current and low-frequency signal noise integrated over a short period, subtracting *S*_OFF_ from *S*_ON_ removes the photodiode dark current steady component and low-frequency noise from the actual light measurement signal. This allows for precise measurements, up to femtoampere precision [21]. By controlling the integration time, the measurement sensitivity could be adjusted. We modified the original circuit design to accommodate an optocoupler to control the LED instead of the built-in LED driver circuit. This modification enabled the use of different LED setups during testing. Since the initial light measurements of the test subjects varied, the photometer was calibrated with a matching integration time at the beginning of each experiment. All optical components were positioned within the incubator and operated for a minimum of 20 min before the experiment to ensure the thermal stability of the photodiodes and LEDs throughout the study. The integration time of the photometer was calibrated at the start of each experiment to accommodate differences in the baseline light intensity measurements due to different hematocrit values between different blood samples. Following calibration, the measurements were recorded continuously until termination. The data were subsequently processed.

To account for fluctuations in light intensity at the beginning of an experiment, light intensity at t = 100 s was set as the baseline. To determine relative light intensity, each measurement of light intensity measured after 100 s was divided by the light intensity recorded at the 100 s time point.

### 2.4. Experimental Groups

The initial experiments were carried out using different concentrations of heparin to establish a setup that would allow adequate anticoagulation to prevent immediate clotting while still allowing blood clots to form within 120 min.

Two experimental groups were defined: a clotting group with induced blood clot formation and a control group without blood clot formation. A heparin concentration of 0.6 IE/mL was used for the experiments with blood clot formation (clotting group). Conversely, for the experiments without blood clot formation (control group), a heparin concentration of 1.5 IE/mL blood was used (control group).

### 2.5. Experiment Duration and Criteria for Experiment Termination

Based on the preliminary experiments, a total duration of 120 min was predetermined for each experiment, allowing for a specific time window to observe blood clotting. If blood clot formation did not occur 90 min after the initiation of the experiment, thrombogenesis was accelerated by adding protamine to the test circuit. The experiment was terminated once a consistent pressure difference of ΔP > 100 mmHg was observed. The selection of this cutoff adheres to the criteria for an acute change in a clinical setting, i.e., a rise in the pressure difference across the membrane oxygenator [6] indicating the formation of a blood clot. Once the experiment was terminated, a final blood sample was collected from the circuit, and the cuvette was rinsed with sodium chloride. The test chamber was illuminated from the bottom using a flashlight to visualize the blood clot.

### 2.6. Blood Sampling and Analysis

During the experiments, blood samples were collected every 10 min into a 1.1 mL serum tube (41.15000.05, Sarstedt, Nümbrecht, Germany) and a 1 mL citrated tube (41.1506, Sarstedt, Nümbrecht, Germany). Blood samples were analyzed using an automated cell counter (MEK-6500K, Nihon Kohden, Tokyo, Japan).

After collecting the blood samples, they were centrifuged at 2000 g for 10 min at room temperature (21 °C). The samples were then aliquoted to obtain both blood plasma and serum samples. These samples were stored at −80 °C. The blood serum samples were used to perform an enzyme-linked immunoassay (SEA370Hu 96 Kit for Beta-Thromboglobulin, Cloud-Clone Corp., Katy, TX, USA) to obtain a quantitative measurement of β-TG. This measurement represents an additional proof of blood clot formation.

### 2.7. Statistical Analysis

The statistical analysis was performed using GraphPad Prism software (Version 9.3.1, GraphPad Software, San Diego, CA, USA). Normal distribution was tested using the Shapiro–Wilk test. Normally distributed data were analyzed using one-way ANOVA. Nonparametric data were analyzed using a Kruskal–Wallis test.

Since blood clotting occurred at different time points in each experiment, the 25 min interval prior to the significant increase in pressure difference was analyzed. The mean values of each time point within this interval were compared to the baseline. A one-way ANOVA and a Kruskal–Wallis test were used to analyze the FSL intensity and pressure data.

A two-way-ANOVA (Šídák’s multiple comparisons test) was used to analyze the ELISA data, specifically comparing the beta-thromboglobulin (β-TG) concentration in the clotting group with that of the control group.

PLT count data were also analyzed using two-way ANOVA (Šídák’s multiple comparisons test). Statistical significance was defined as *p* < 0.05. Unless specified otherwise, all data are expressed as the mean ± standard error of the mean (SEM).

## 3. Results

### 3.1. Blood Clot Formation

**Figure 3:**
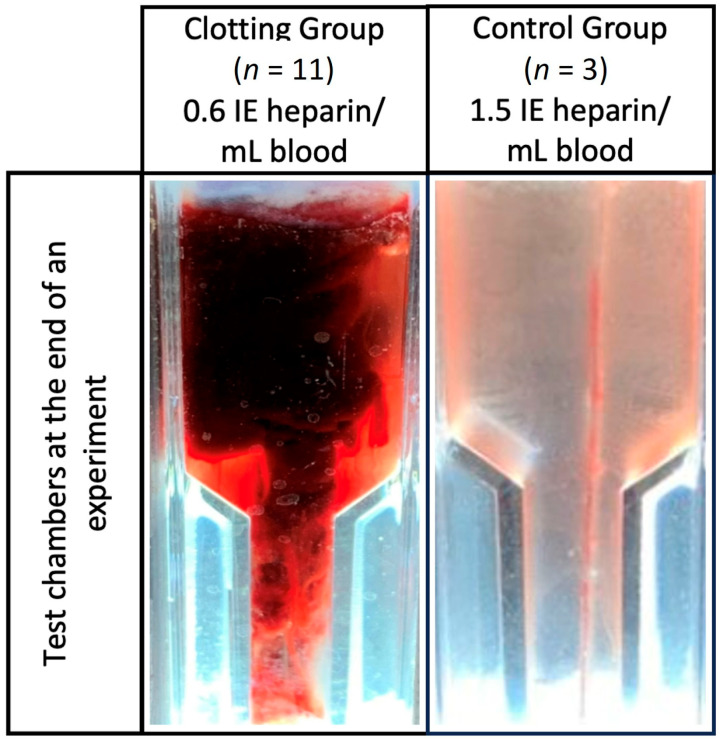
Test chambers at the end of an experiment and after flushing with sodium chloride.

In 11 experiments, blood clot formation occurred (Figure 3). In these experiments, the heparin concentration was 0.6 IE/mL blood. In the control group (*n* = 3), no blood clot formation occurred after 90 min. This group used a heparin concentration of 1.5 IE/mL blood (Table 1).

### 3.2. FSL and Pressure Measurements

**Figure 4:**
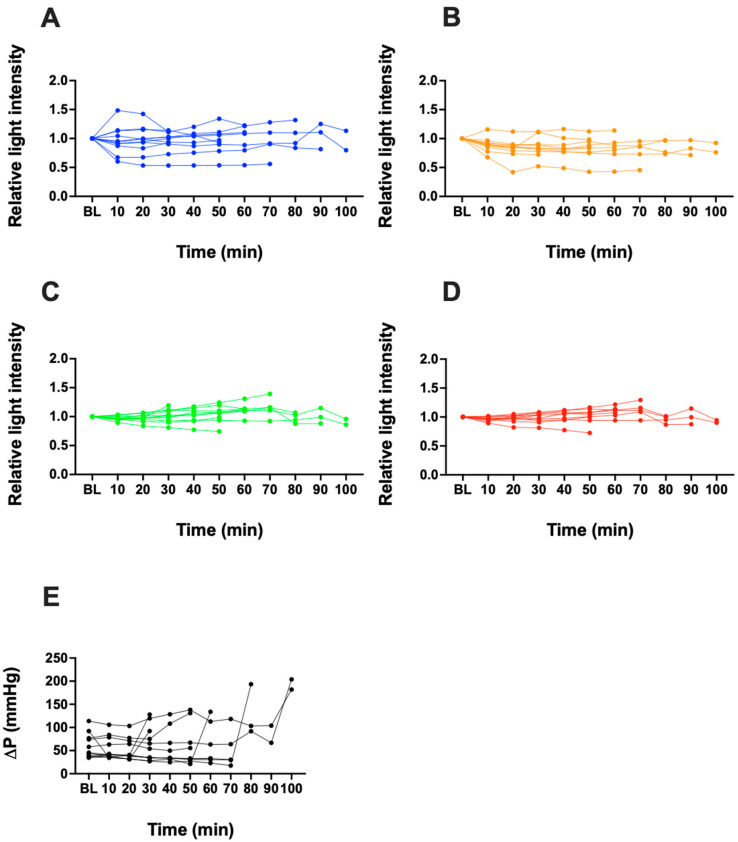
Measurement of relative light intensity of FSL from LED 1 (**A**), LED 2 (**B**), LED 3 (**C**), and LED 4 (**D**) over time until fulminant blood clot formation (*n* = 11). Measurements of pressure (ΔP) over time until fulminant blood clot formation is shown in (**E**) (*n* = 11).

**Figure 5:**
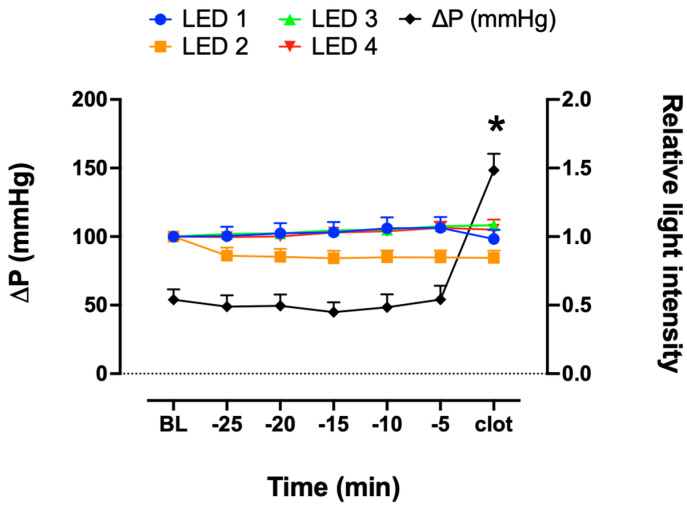
Measurement of relative light intensity of FSL from LEDs 1, 2, 3, and 4 and pressure measurements (ΔP) 25 min before blood clot formation (*n* = 11). All data are presented as mean ± SEM. * *p* < 0.05 versus baseline, Kruskal–Wallis test.

**Figure 6:**
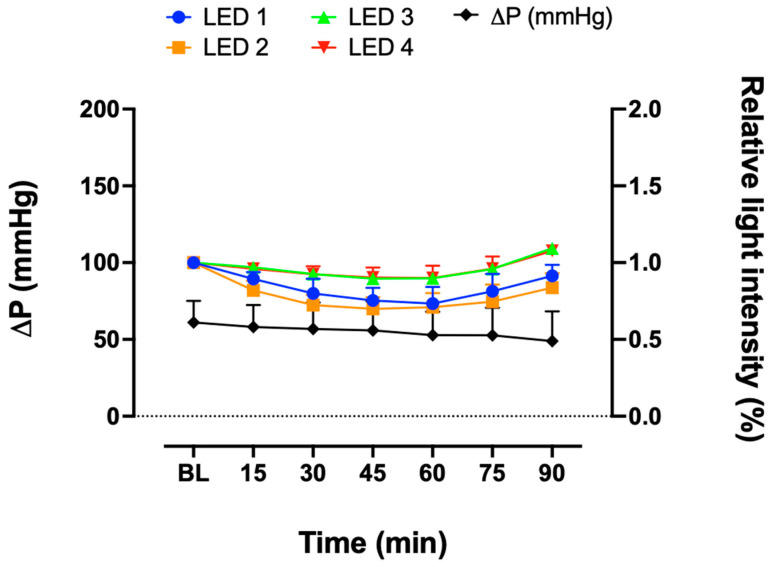
Measurement of relative light intensity of FSL in the control group from LEDs 1, 2, 3, and 4 and measurement of pressure difference (ΔP) over time (*n* = 3). Kruskal–Wallis test and one-way ANOVA; mean ± SEM.

In each experiment, blood clot formation occurred at different time points (Figure 4). Therefore, the data were compared in a timeframe of 25 min before the blood clot formation. There was no significant change in FSL measurements at LED 1, 2, 3 or 4 when compared to the baseline of each LED (Figure 5). Pressure increased at the end of the experiments by the time a fulminant clot occurred, which defined the termination of the experiments (* *p* < 0.05 versus baseline). In contrast, no significant change in the signal was detected for any of the LEDs. In the control group, neither the FSL nor pressure measurements changed over time (Figure 6).

### 3.3. Platelet Count and Beta-Thromboglobulin

**Figure 7:**
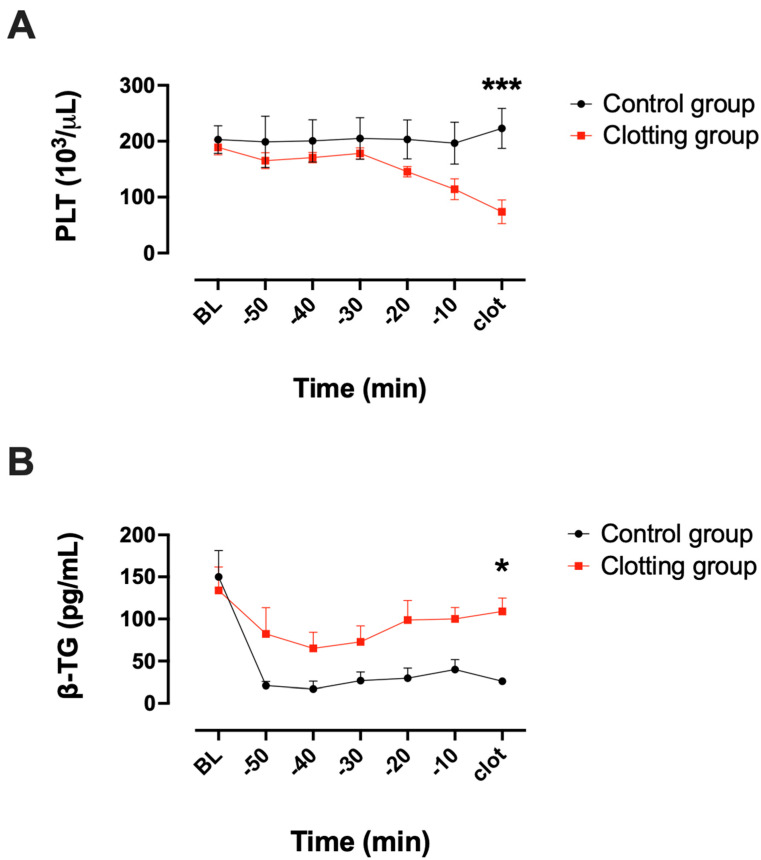
(**A**) Platelet (PLT) counts 50 min before fulminant blood clot formation in the blood clotting group (*n* = 11) and in the control group (*n* = 3). (**B**) β-TG concentrations before blood clot formation in the blood clotting group (*n* = 9) and in the control group (*n* = 3). Two-way ANOVA; mean ± SEM; * *p* < 0.05, *** *p* < 0.001.

The concentration of PLTs significantly decreased at the end of the experiment in the clotting group (t = clot; *** *p* < 0.001 vs. control group) (Figure 7A). There was no significant change in the PLT count in the control group. The concentration of β-TG was significantly higher at blood clot formation (t = clot) compared to that of the control group (* *p* < 0.05) (Figure 7B), whereas the concentration of β-TG at baseline was not significantly different between the two groups.

## 4. Discussion

This study aimed to assess the potential of using FSL measurements for early blood clot detection within a blood-filled test chamber of an in vitro test circuit and to compare the results to ΔP measurements, the current clinical gold standard for blood clot detection in an ECMO device.

We induced fulminant blood clot formation within the in vitro circuit, shown by a significant increase in ΔP and a decrease in the PLT count in the clotting group. In contrast, the control experiments showed no blood clot formation or alterations of the mentioned parameters. Contrary to our expectations, we found that the measurement of FSL was not superior to the measurement of ΔP across the test chamber.

Since the absorbance of blood is determined by the absorbance of Hb, 850 nm LEDs were selected owing to the low absorbance of this wavelength by Hb and, accordingly, their high transmittance in blood [22]. Previous studies used LEDs at the same wavelength [18]. Other studies used laser light instead of LEDs as a light source [23,24]. Lasers offer the benefit of producing light at a specific, narrow wavelength, whereas LEDs, by contrast, emit light in a broader range around a chosen wavelength. This distinction is crucial in applications where precision in light wavelength is paramount. The disadvantages of lasers are that they are large, require much space, and are expensive; therefore, they are challenging to implement in the clinic. In contrast, LEDs are small, cheap, and easy to implement.

A retrospective analysis demonstrated that in cases requiring urgent ECMO system replacement, blood clot formation within the membrane oxygenator accounted for 19% of exchanges [15]. To ultimately assess a blood clot formation within an ECMO device, we decided to measure the light intensity of FSL as opposed to other studies that measured BSL intensity [18,25]. In these studies, the light source and the photodiodes were positioned on the same side, measuring the reflection of light on the surface of the part of the ECMO circuit where blood clot formation was expected to occur. One disadvantage of this approach is that BSL can only detect blood clots on the surface of a blood pump or an ECMO device. Deeper blood clots remain undetected. In the studies that used FSL for blood clot detection [17,24,26], the LEDs were either placed on clear tubing or a blood pump head. The blood layer thickness did not exceed 5 mm [18]. In this study, we employed a test chamber with an optical path distance twice as long as previously reported (10 mm). In another approach, one might consider detecting a blood clot in the ECMO device by using side-scattered light instead of or in addition to BSL or FSL. Since light is also scattered sideways, it might increase the likelihood of detecting a clot within the “ECMO device”.

Prior studies used blood flow rates up to 6 L/min within the ECMO test circuit [25]. As an intended proof-of-principle study, we decided to use a miniaturized test circuit with a low priming volume and a low blood flow rate. It has been shown that the blood flow rate affects the composition of a thrombus. While venous thrombi primarily consist of fibrin and red blood cells (“red blood clots”), arterial thrombi usually develop under high blood flow conditions. They are mainly composed of PLT aggregates, resulting in their appearance as white blood clots [27].

Finally, the measurement of relative light intensity is subject to significant fluctuations due to factors such as hematocrit, oxygen saturation, osmolarity, and hemolysis, which might also account for the differences seen between this and prior studies [28].

When blood comes into contact with an artificial surface, blood clotting is promoted through a complex cascade that includes plasma protein adsorption [29,30,31]; adhesion of PLTs, leukocytes, and red blood cells [32,33]; thrombin generation; and complement activation [34,35]. Plasma proteins such as von Willebrand factor (vWF) and fibrinogen are adsorbed to the artificial surface and mediate the attachment of PLTs, reducing the overall PLT count. A significant reduction in PLT concentration was found to correlate with thrombus formation in an ECMO circuit [36,37]. In this study, the concentration of PLTs significantly dropped at the end of the experiment, indicating fulminant blood clot formation. No significant change in the PLT count was detected in the control group.

β-TG is a protein stored in PLTs and released when they are activated by various agents, including thrombin [38,39]. β-TG has been demonstrated to be a helpful marker of PLT activation [40,41]. A normal level of β-TG indicates that increased PLT activation did not occur [42]. In this study, the concentration of β-TG was significantly higher in the clotting group compared to the control group at the end of the experiments indicating a lower heparin effect and a stronger tendency towards clot formation.

### Limitations

This study has some limitations. Firstly, we only used a miniaturized in vitro test circuit instead of an ECMO circuit with a larger priming volume and a higher blood flow rate, such as those used in clinical settings.

Secondly, we did not use an ECMO device with gas exchange membranes in this in vitro circuit. Gas exchange membranes might diminish the intensity of the FSL. As an intended proof-of-principle study, a simplified approach with a blood-filled test chamber was used instead.

## 5. Conclusions

In conclusion, this study showed that the measurement of FSL at a wavelength of 850 nm is not superior to ΔP measurements. Future studies should investigate if FSL at other wavelengths will induce signal differences as blood clotting occurs and if this technique is transferable to multilayer oxygenator membranes.

## Figures and Tables

**Table 1:** Overview of clotting group (*n* = 11) and control group (*n* = 3). Sex of blood donor (m = male; f = female), hematocrit (HCT), and occurrence of blood clot formation are shown.

		Sex of Blood Donor	HCT [%]	Blood Clot Formation?
Clotting Group (*n* = 11) 0.6 IE heparin/mL blood	1	m	46.4	Yes
	2	m	44	Yes
	3	m	43.5	Yes
	4	m	43.2	Yes
	5	m	44.3	Yes
	6	m	46.3	Yes
	7	f	38.9	Yes
	8	m	45.6	Yes
	9	m	44.9	Yes
	10	m	41.4	Yes
	11	f	36.2	Yes
Control Group (*n* = 3) 1.5 IE heparin/mL blood	12	f	38.9	No
	13	f	36.3	No
	14	f	43.2	No

## Data Availability

The datasets generated and/or analyzed during the current study are available from the corresponding author upon reasonable request.
